# Kidney and Pregnancy: A Comprehensive Review

**DOI:** 10.3390/clinpract15100189

**Published:** 2025-10-20

**Authors:** Luca Piscitani, Paolo Sipari, Lorenzo Ottavio Di Pietro, Sofia Bussolaro, Maurizio Guido, Ilaria Fantasia

**Affiliations:** 1 Nephrology and Dialysis Unit, Medical Department, San Salvatore Hospital, via Vetoio 1, 67100 L’Aquila, Italy; lpiscitani@asl1abruzzo.it (L.P.); psipari@asl1abruzzo.it (P.S.); lodipietro@asl1abruzzo.it (L.O.D.P.); 2 Obstetrics & Gynaecology Unit, San Bassiano Hospital, 36061 Bassano del Grappa, Italy; sofia.bussolaro91@gmail.com; 3 Department of Life, Health and Environmental Sciences, University of L’Aquila, 67100 L’Aquila, Italy; maurizioguido@libero.it; 4 Department of Pharmacy and Health and Nutrition Sciences, Università Della Calabria UNICAL, 87036 Cosenza, Italy; 5 Obstetrics & Gynaecology Unit, San Salvatore Hospital, 67100 L’Aquila, Italy

**Keywords:** pregnancy, kidney disease, HELLP syndrome, thrombotic microangiopathy, systemic lupus erythematosus

## Abstract

During pregnancy, a series of physiological changes occur in women, particularly affecting the cardiovascular system with significant hemodynamic alterations. Subsequently, this leads to renal adaptations manifesting through variations in glomerular filtration rate. This close interconnection between the heart and kidneys implies that issues arising in one organ will disrupt this fundamental balance, inevitably involving all associated organs. The purpose of this review is to gather all possible nephrological conditions that may arise during pregnancy, as well as pre-existing conditions that may become apparent or worsen during this period. This review describes the natural history, treatment, and impact of these conditions on pregnancy itself. Among the most common conditions are preeclampsia and HELLP syndrome, severe complications characterized by hypertension, proteinuria, and multiorgan damage that require immediate clinical attention. Additionally, women with chronic kidney disease are at higher risk of developing maternal–fetal complications, such as preterm birth and intrauterine growth restriction. Common causes of acute renal failure are also analyzed, including thrombotic microangiopathy, acute fatty liver of pregnancy, acute onset or flare of systemic lupus erythematosus, and catastrophic antiphospholipid antibody syndrome. Given the importance of proper renal function during pregnancy, it is essential to have a thorough understanding of nephrological diseases that may affect this phase of women’s lives. This knowledge is crucial for managing these conditions effectively to avoid risks to the survival of both the mother and the newborn.

## 1. Introduction

Approximately 3% of women of childbearing age suffer from renal function deficiency and/or albuminuria [1,2]. The presence of kidney function impairment can lead to severe pregnancy complications, whose incidence and severity are more serious the greater the stage of nephropathy [3]. The aim of this comprehensive review is to illustrate the most common kidney diseases in pregnancy, both primary and pregnancy-induced, discussing natural history, management, and impact on the course of pregnancy.

## 2. Physiologic Changes in Pregnancy

Pregnancy induces significant physiological changes in a woman’s body, and the kidneys are no exception. These changes are necessary to support the increased demands of the mother and the developing fetus. During pregnancy, not only does a progressively noticeable hemodynamic change occur at the cardiac level with an increase in stroke volume, heart rate, and consequently also in cardiac output by 20–40%, but also renal blood flow and glomerular filtration rate (GFR) increase by about 50% [4]. These adaptations are crucial for managing the increased blood volume and metabolic waste from the fetus [5]. Because of the interconnection between cardiac and renal function, a dysfunction of the body’s adaptation mechanisms to pregnancy can produce cardiovascular alterations, causing in turn an alteration in renal function and vice versa. The normal serum creatinine level in pregnancy is in the range of 0.4 to 0.6 mg/dL. This enhancement begins in the first trimester, peaks around mid-pregnancy, and is maintained until term [5]. The hormonal influences, particularly the hormone relaxin, play a key role in mediating these changes. Relaxin induces vasodilation, leading to reduced renal vascular resistance and increased renal plasma flow [6]. The kidneys undergo adaptations in handling solutes to maintain homeostasis. For instance, while the GFR increases, the renal threshold for glucose decreases, which can result in mild gestational glycosuria [7]. Additionally, there is increased sodium retention to support the expanded blood volume, partly due to increased aldosterone levels [8]. Urine protein excretion increases during normal pregnancy, from 60–90 mg/dL to 180–250 mg/dL, as measured by a 24 h urine collection. This is a consequence of hyperfiltration, changes in glomerular permeability, and an increase in tubular proteinuria. Finally, there is a change in the immune system during pregnancy with a shift from a T helper (TH) type 1 (TH1; cell-mediated immunity) to a TH-2 cell (humoral-mediated immunity) to ensure greater fetal tolerance. This may predispose to the onset or recurrence of an autoimmune disease.

Understanding these adaptations and their clinical implications is crucial for managing renal health during pregnancy, for the diagnosis of new-onset kidney diseases, and for monitoring those with pre-existing conditions.

## 3. Urinary Tract Infection

In pregnancy, UTIs form a spectrum; progression to pyelonephritis directly involves the kidney and drives adverse outcomes, justifying inclusion in a review of kidney disease in pregnancy.

A urinary tract infection (UTI) during pregnancy is very common but treatable. It occurs when bacteria, typically Escherichia coli from the digestive tract, enter the urinary system and multiply in the bladder. Pregnant women are particularly susceptible due to pregnancy-induced changes in their bodies [9]. Indeed, predisposition during pregnancy to UTI is secondary to hormonal changes, especially progesterone, which can cause the ureteral muscles to relax. This relaxation can slow down the flow of urine, making it easier for bacteria to multiply [10]. In addition, the uterus, as it grows, can compress the bladder and urinary tract, causing incomplete emptying of the bladder and dilation of the ureters [11]. Proper hygiene and correct hydration are fundamental to preventing UTIs, while early detection and treatment are crucial to prevent complications [12]. Regular prenatal care, including urine screenings, can help ensure both the mother’s and baby’s health. The main symptoms are pollakiuria, burning urine, cloudy urine with an intense smell, pain, and discomfort in the lower abdomen with fever. If untreated or inadequately treated, UTIs can lead to more serious infections, such as pyelonephritis and obstetrical sepsis, even though the most common manifestation is cystitis. These last conditions can be dangerous for both the mother and the fetus and may lead to preterm labor or low birth weight [13].

Asymptomatic bacteriuria occurs in 2–7% of pregnant women, similarly to non-pregnant women, with a higher prevalence in diabetic women, 8–14% [14]. It is important to screen for this condition during prenatal visits to prevent potential complications, such as preterm labor (aRR 1.3) [15]. Screening for asymptomatic bacteriuria should be performed between 12 and 16 weeks of gestation (or at the first antenatal visit if this occurs later). If negative, it should be repeated at the beginning of the third trimester. If positive, antibiotic treatment lasting approximately 7 days should be started. The antibiotic regimen should be based on the results of the antibiogram. However, the preferred antibiotics are fosfomycin and beta-lactams (cephalosporins, penicillin) with or without beta-lactamase inhibitors. Other antibiotics with a good safety profile in pregnancy are erythromycin, azithromycin, and clindamycin [16]. Aminoglycosides, tetracyclines, and fluoroquinolones should be avoided during pregnancy and lactation.

## 4. Hypertensive Disorders of Pregnancy

Hypertensive disorders of pregnancy (HDPs) are common, occurring in 6% to 8% of pregnancies [17]. Hypertension during pregnancy is a significant cause of maternal and fetal morbidity and mortality. Understanding the risk factors, management strategies, and outcomes is crucial for improving both maternal and fetal health.

Hypertension in pregnancy should be diagnosed when systolic blood pressure is ≥140 mmHg and/or diastolic blood pressure is ≥90 mmHg. If hypertension occurs after the 20th week of gestation, it will be classified as gestational hypertension; otherwise, if it is found before the 20th week, it will be classified as pre-gestational or chronic hypertension [18]. New-onset hypertension after 20 weeks plus proteinuria (≥300 mg/24 h or protein/creatinine ≥ 0.3; dipstick ≥ 2+ if no quantitative test) or new-onset maternal organ dysfunction (e.g., thrombocytopenia, renal insufficiency, impaired liver function, pulmonary edema, or cerebral/visual symptoms) with or without proteinuria is diagnostic for preeclampsia [19]. Hypertension alone after 20 weeks of gestation may be the first sign of preeclampsia, especially if it is associated with proteinuria [20]. Superimposed preeclampsia should be considered at the onset of significant proteinuria ≥ 20 weeks in a patient diagnosed with chronic hypertension [21]. The spectrum of HDP includes gestational hypertension, chronic hypertension, preeclampsia, and preeclampsia superimposed on chronic hypertension. Although preeclampsia is triggered by placental pathology, its maternal phenotype is characterized by systemic endothelial injury with prominent renal involvement (proteinuria, reduced GFR), which places it squarely within the scope of pregnancy-related kidney disorders.

### 4.1. Gestational Hypertension

Gestational hypertension, also known as pregnancy-induced hypertension (PIH), is a condition characterized by the presence of elevated blood pressure without proteinuria or multiorgan dysfunction. The exact cause of gestational hypertension remains unclear. However, risk factors have been identified, including advanced maternal age, obesity, multiple pregnancy, and pre-existing medical conditions like diabetes or chronic kidney disease. Gestational hypertension is usually asymptomatic, and its diagnosis follows the finding of BP values ≥ 140/90 mmHg. Laboratory tests are performed mainly to rule out preeclampsia. Proteinuria, defined by the excretion of >300 mg of protein in a 24 h urine collection, is the main criterion for making a differential diagnosis. Secondarily, platelet count, serum creatinine, and liver function can be requested. The prognosis of women with gestational hypertension is usually favorable; however, progression to preeclampsia can occur in 10–50% of patients with gestational hypertension [22]. Antihypertensive medication should be started at diagnosis (systolic blood pressure is ≥140 mmHg or diastolic blood pressure ≥ 90 mmHg) [23]. Antihypertensive medications like nifedipine (calcium channel blockers), labetalol (beta-blocker), and methyldopa are commonly used based on the patient’s individual characteristics. Patients with gestational hypertension can have an outpatient management. However, they need to be educated on signs and symptoms suggestive of preeclampsia, like headache, visual disturbance, epigastric or high-quadrant abdominal pain. A blood pressure home diary is also advised.

### 4.2. Preeclampsia and Eclampsia

Preeclampsia is a multisystemic disorder that can seriously affect pregnancy outcome. Eclampsia is the extreme expression of preeclampsia and is defined as the occurrence of one or more convulsive episodes, which have no other known origins. Although the etiology and pathophysiology are not yet well understood, phenomena of placental hypoperfusion caused by altered angiogenetic factors, immune system maladaptation with an exaggerated inflammatory response, and increased sensitivity to angiotensin II, which collectively lead to systemic endothelial dysfunction and vasospasm, reducing renal perfusion and GFR, would seem to be involved [24]. Additional factors such as obesity and metabolic disorders, gestational diabetes, multiple pregnancies, and certain maternal–fetal and autoimmune diseases appear to increase the baseline risk [25]. If not managed promptly, preeclampsia can progress to eclampsia, which significantly jeopardizes both maternal and fetal health. The increase in serum creatinine may be small, but it should be compared to the creatinine values taken at the beginning of the pregnancy, which are reduced due to hyperfiltration. Due to the above pathogenesis and generalized edema, oliguria may develop, complicating preeclampsia and its treatment. Because of the risk of complicating into pulmonary edema, both intravenous and oral fluids should be limited, and for treatment of persistent oliguria, furosemide and diuretics are not first-line antihypertensive drugs in pregnancy due to the potential risk of reduced uteroplacental blood flow and maternal electrolyte imbalance. However, they could be indicated when pulmonary edema is present. Also, low-dose dopamine should not be used because of the absence of clear scientific evidence. Some other drugs, like ACE inhibitors and angiotensin receptor blockers (ARBs), should be avoided as they carry an increased risk of malformations, growth retardation, oligohydramnios, and fetal anuria [26]. The treatment for preeclampsia is limited and involves antihypertensive medications, which include first-line and second-line medications according to published guidelines [20,21]. Magnesium sulfate is indicated for seizure prevention and neuroprotection of the mother and the fetus, and delivery when indicated to prevent disease progression. New scientific evidence has shown that in hypertensive disorders, it is possible to stratify the risk for adverse pregnancy events and determine the most accurate treatment for each case, depending on maternal hemodynamics and cardiovascular profile, studied using non-invasive methods [27]. It would appear that tailored treatment with the aforementioned drugs can correct the underlying maternal hemodynamic imbalance and prevent or mitigate incipient preeclampsia and its complications [28].

A recent important development in the management of preeclampsia has been the introduction of strategies of first-trimester screening and prevention of the disease. Women at high risk of developing preterm preeclampsia (≤37 weeks) are identified through a first-trimester screening test incorporating maternal characteristics, mean arterial blood pressure, uterine artery pulsatility index, and maternal serum analytes, such as pregnancy-associated plasma protein-A (PAPP-A) and placental growth factor (PlGF) [29]. This method is able to identify 82% of women at risk of developing preterm preeclampsia [30]. The importance of this approach lies in the possibility of performing an effective drug prevention based on aspirin 150 mg/day to be taken from 11–14 weeks until 36 weeks of gestation, supported by the evidence from the ASPRE trial [31]. This screening and prevention strategy allows for a reduction in the rate of delivery with preterm preeclampsia by 62%.

## 5. Acute Kidney Injury During Pregnancy

Acute kidney injury (AKI) in pregnancy, although relatively rare, can arise from various causative factors. Acute renal failure occurring in pregnancy or postpartum is part of a very different set of conditions. Although they have different etiological and pathogenetic mechanisms, they share pathophysiological abnormalities, clinical manifestations, and laboratory data, making differential diagnosis difficult. The shared pathophysiological abnormalities are vasoconstriction, activation, and consumption of platelets, microangiopathic intravascular hemolysis, microcirculation thrombosis, endothelial dysfunction, and reduced tissue perfusion. The onset of AKI during pregnancy is critical as it is associated with an increased risk of maternal and fetal morbidity and mortality, including an increased risk of maternal death (OR 4.5), disseminated intravascular coagulation (3.4), placental abruption (3.1), and neonatal death (3.4) [32]. Emerging biomarkers may offer utility in the early diagnosis of AKI and provide insight into its underlying pathophysiological mechanisms, such as the following:

- Neutrophil Gelatinase-Associated Lipocalin (NGAL): an early marker of acute tubular injury;
- Kidney Injury Molecule-1 (KIM-1): expressed by proximal tubular epithelial cells and useful in the early detection of acute kidney injury;
- Cystatin C: an endogenous inhibitor of cysteine proteases, shown to estimate glomerular filtration rate (GFR) more sensitively than serum creatinine.

Currently, there is insufficient empirical evidence to support the routine clinical use of these biomarkers, with the exception of Cystatin C. However, its levels may be influenced by placental production during pregnancy [33].

Forms of AKI that may develop during pregnancy include preeclampsia and eclampsia, discussed above, HELLP (H—hemolysis, EL—elevated liver enzyme levels, LP—low platelet levels) syndrome, thrombotic microangiopathy, acute fatty liver of pregnancy, acute onset or flare of systemic lupus erythematosus (SLE), and catastrophic antiphospholipid antibody syndrome. Management of the most frequent causes of AKI is reported in the following paragraphs and summarized in Table 1.

### 5.1. HELLP Syndrome

HELLP syndrome is a serious condition that manifests itself more insidiously than the other, characterized by hemolysis, elevated liver enzymes, and low platelet count that can precipitate in AKI [34]. Although the pathophysiological mechanisms behind HELLP syndrome remain poorly understood, several hypotheses have been proposed:

- Etiological overlaps with thrombotic microangiopathy, involving microvascular endothelial activation, cell damage, and subsequent thrombosis;
- Immune-mediated fetal rejection;
- Placenta-driven hepatocyte apoptosis mediated by the CD95/CD95-L (Fas/Fas ligand) pathway;
- Genetic abnormalities in fatty acid metabolism;
- Involvement of platelet plasminogen pathways [35].

The risk of maternal mortality is around 1% due to possible evolution into disseminated intravascular coagulation, placental abruption, pulmonary edema, acute renal failure, liver failure, hepatic hemorrhage, acute respiratory distress syndrome, and stroke. If renal failure develops, differential diagnosis with other syndromes, especially pregnancy-associated hemolytic uremic syndrome (HUS), is essential. Cases of unpreserved diuresis and dialysis requirements are rare. In this case, renal biopsy could document acute tubular necrosis and signs of thrombotic microangiopathy [36].

### 5.2. Thrombotic Microangiopathy

Thrombotic microangiopathy refers to a pathologic process in which arterioles and capillaries are involved, leading to microvascular thrombosis, microangiopathic hemolytic anemia, and thrombocytopenia. The microangiopathies include the thrombotic thrombocytopenic purpura (TTP) and the HUS. Both syndromes can result in AKI due to microvascular thrombosis. Physiological pregnancy may trigger the onset or recurrence of the TTP mainly in the latter part of the second and third trimester, because of a physiological reduction (reduced production or effect of inhibitors) in ADAMS13 and a progressive increase in von Willebrand factor. TTP requires plasma exchange that removes ADAMTS13 inhibitors from the circulation. HUS is invariably associated with AKI. It has a severe prognosis: about 75% of patients require replacement treatment one month after diagnosis. Treatment might include eculizumab, which is a humanized monoclonal antibody functioning as a terminal complement inhibitor. This drug is administered by intravenous infusion. Its pharmacological effect is accomplished by binding to the terminal complement component 5, or C5, and blocks the cleavage of C5 into C5a and C5b, both of which have potent prothrombotic and proinflammatory effects. Eculizumab may be regarded as safe in pregnancy; it is not present in breast milk, and its levels observed in umbilical cord blood samples are not sufficient to affect the concentration of complement in newborns [37].

### 5.3. Acute Fatty Liver of Pregnancy

Acute fatty liver of pregnancy (AFLP) is a rare but severe condition that can lead to acute liver failure and subsequent renal impairment. Renal failure is part of the hepato-renal syndrome. Acute fatty liver of pregnancy (AFLP) is characterized by microvesicular steatosis of the liver, which is believed to result from mitochondrial dysfunction in the β-oxidation of fatty acids. This impairment leads to the accumulation of fatty acids within hepatocytes. Prompt delivery of the fetus remains the cornerstone of treatment in AFLP, irrespective of gestational age.

Ascites can be present or related in pregnancy to a previous liver disease. Management primarily focuses on treating the underlying cause. General measures include the following:

- Dietary sodium restriction, because reducing sodium intake can help decrease fluid accumulation;
- Diuretics: in particular, spironolactone is often the preferred one due to its safety profile in pregnancy, even if close monitoring is essential;
- Paracentesis is typically considered when conservative measures fail and can be proposed to relieve symptoms in severe cases;
- Managing the underlying causes, for example, addressing liver dysfunction, optimizing cardiovascular health, and closely monitoring renal function [38].

However, the cornerstone of AFLP management is prompt delivery of the fetus, irrespective of gestational age, as maternal condition typically improves postpartum. Supportive care in a high-dependency or intensive care setting is essential and includes correction of hypoglycemia with intravenous glucose, management of coagulopathy with blood products, renal support in cases of acute kidney injury, and treatment of hepatic encephalopathy. Multidisciplinary involvement—encompassing obstetrics, hepatology, critical care, and nephrology—is recommended. Postpartum, maternal recovery should be closely monitored, and the neonate should be screened for fatty acid oxidation disorders such as long-chain 3-hydroxyacyl-CoA dehydrogenase (LCHAD) deficiency. Genetic counseling may be considered in selected cases.

### 5.4. Acute Onset or Flare of Systemic Lupus Erythematosus

Systemic lupus erythematosus (SLE) may begin in pregnancy and mimic the picture of severe preeclampsia, especially in the second and third trimesters. Pregnancy can increase the risk of lupus flares, especially if the disease is active in the six months before conception [39]. There is an increased risk of miscarriage, preterm birth, and intrauterine growth restriction (IUGR) in pregnant women with lupus [40]. Rarely, babies born to mothers with SLE may have neonatal lupus, which can cause a temporary rash and/or liver problems. In some cases, it can lead to congenital heart block [41]. Hydroxychloroquine and azathioprine are considered safe and often used because of no known risk for teratogenicity. Glucocorticoids can be used, but the risk of gestational diabetes, cleft lip and palate, and premature rupture of membranes should be mentioned to the patient. Methotrexate and cyclophosphamide should be avoided [42]. The adverse effects of Rituximab in pregnancy are not known, but it may cause transient B-cell depletion in the fetus.

### 5.5. Catastrophic Antiphospholipid Antibody Syndrome

Catastrophic antiphospholipid antibody syndrome (CAPS) is a life-threatening condition, a dreaded evolution of the antiphospholipid antibody syndrome [43]. It is characterized by multiple disseminated thrombosis, with extensive parenchymal involvement, leading to rapidly progressive multi-organ failure (MOF). It starts early during pregnancy. Early miscarriages and pregnancy failures may result from a CAPS.

## 6. Chronic Kidney Disease

Chronic kidney disease (CKD) poses significant risks for a pregnant woman and her fetus. The interaction between CKD and pregnancy status is a sensitive topic, especially regarding maternal–neonatal outcomes. CKD can complicate pregnancy, and even pregnancy itself can worsen renal function. In both cases, the development of complications that can worsen maternal–neonatal outcomes is possible. Recent studies highlight the increased risk of adverse maternal outcomes in pregnant women with CKD because of a higher incidence of preeclampsia, gestational hypertension, and preterm delivery [44]. The degree of renal impairment plays a significant role in determining the severity of these outcomes. Women with advanced CKD stages (stage III–V) experience more complications compared to those in the early stages (stage I–II). Hladunewich et al. found that women with CKD have a 30–40% increased risk of preeclampsia [45]. This risk is further amplified in those with significant proteinuria. Likewise, the impact of maternal CKD on fetal outcomes has been a major focus. Studies show that CKD increases the risk of IUGR, low birth weight, and neonatal intensive care unit (NICU) admissions. There is also a higher incidence of stillbirths and neonatal mortality in women with severe CKD. Piccoli et al. demonstrated that babies born to mothers with CKD have a 2.5 times higher risk of being born preterm and a 3.5 times higher risk of having low birth weight [46]. For this reason, multidisciplinary approaches involving nephrologists, obstetricians, neonatologists, and other healthcare professionals lead to better outcomes.

Early identification and management of potential complications are crucial. Tangren et al. emphasized that close monitoring and tailored interventions significantly reduce the risk of adverse outcomes in pregnant women with CKD. Managing CKD during pregnancy involves balancing the progression of renal disease with the health of the mother and fetus. Recent guidelines recommend optimizing blood pressure control, managing proteinuria, and closely monitoring renal function. Bramham et al. suggested that optimizing medication for blood pressure control can improve both maternal and fetal outcomes [47].

Postpartum care is critical as well as the pregnant period for women with CKD, as they are at risk for rapid deterioration in kidney function. Monitoring and managing hypertension, glucose levels, and renal function are necessary to mitigate long-term effects on kidney health. Postpartum follow-up within the first six weeks significantly helps in early detection and management of renal function deterioration [48].

### Autosomal Dominant Polycystic Kidney Disease

Autosomal dominant polycystic kidney disease (ADPKD) represents the most prevalent monogenic nephropathy and cystic renal disorder, with an estimated prevalence ranging from 1 in 1000 to 1 in 2500 individuals [49]. Current evidence does not support a significant reduction in fertility among women with ADPKD who maintain preserved renal function [50]. The disease frequently manifests during the reproductive years and may be complicated by systemic hypertension, proteinuria, and mild renal insufficiency. The risk of preeclampsia is notably elevated in patients with coexisting chronic kidney disease (CKD), proteinuria, or pregestational hypertension [51]. In line with current obstetric guidelines, low-dose aspirin prophylaxis (81–150 mg daily), initiated before 16 weeks of gestation, should be considered in women with ADPKD who present with CKD, chronic hypertension, or significant proteinuria, given their elevated risk for preeclampsia [52,53]. Management strategies for hypertension and CKD in the context of ADPKD align with standard therapeutic protocols applied to other renal pathologies. Women with a known history of intracranial aneurysm warrant heightened surveillance during pregnancy. Stringent blood pressure control is advised in this cohort, and individualized delivery planning, including mode of delivery, should be considered. Nonetheless, existing literature presents conflicting data regarding an increased incidence of obstetric or neurological complications in these patients [54].

ADPKD is associated with an increased predisposition to urinary tract infections (UTIs) during pregnancy. Approximately 10% of hospital admissions among pregnant women with ADPKD are attributable to infected renal cysts [55]. Tolvaptan, a vasopressin V2 receptor antagonist approved for the treatment of ADPKD, is classified as pregnancy category D and is contraindicated during pregnancy and lactation due to potential teratogenicity and toxicity. Given the autosomal dominant inheritance pattern of ADPKD, offspring of affected individuals carry a 50% risk of disease transmission. Accordingly, preconception genetic counseling is strongly recommended [56].

## 7. Pregnancy in Kidney Transplant Recipients

Pregnancy in women who have undergone kidney transplantation is increasingly common and generally considered feasible with proper management. However, these pregnancies are associated with higher risks for both maternal and fetal complications. The management of pregnancy in kidney transplant recipients requires careful pre-conception counseling, modification of immunosuppressive therapy, and close monitoring throughout the pregnancy to optimize outcomes for both the mother and the child.

Pregnancy after kidney transplantation presents several challenges, primarily due to the need for ongoing immunosuppressive therapy and the potential impact on graft function. Studies indicate that preeclampsia occurs in approximately 20–25% of pregnancies in kidney transplant recipients, significantly higher than the general population, where it occurs in about 3–8% of pregnancies. Additionally, these women are at an increased risk of developing gestational hypertension, with an incidence of up to 50%, particularly in those with pre-existing hypertension or less time elapsed since transplantation [57,58].

Cesarean sections are more common in this population, with rates reported as high as 60–70%, often due to the complexity of managing these high-risk pregnancies. Moreover, while kidney function generally remains stable during pregnancy, there is a risk of graft loss in approximately 1–7%, particularly in those with impaired baseline renal function or acute rejection episodes during pregnancy. However, while pregnancy in kidney transplant recipients may be associated with transient graft dysfunction, the risk of irreversible graft loss remains low in patients with stable allograft function, controlled blood pressure, and appropriate immunosuppression. Careful preconception assessment and multidisciplinary management are essential to optimize maternal, fetal, and graft outcomes.

The outcomes for the fetus are also of concern, with preterm birth rates ranging between 40–50% and low birth weight being common due to both prematurity and intrauterine growth restriction. The live birth rate for pregnancies in kidney transplant recipients is generally favorable, reported at around 73%, but there is still a notable risk of miscarriage (15–20%) and stillbirth (5%) [57].

Immunosuppressive medications, essential to prevent graft rejection, have varying degrees of safety during pregnancy. Calcineurin inhibitors like tacrolimus and cyclosporine are commonly used and have been associated with relatively safe outcomes when dosed appropriately. However, medications such as mycophenolate mofetil are contraindicated due to teratogenicity and must be replaced before conception with other drugs; safer alternatives include azathioprine. Evaluation of the whole immunosuppressive therapy is required before azathioprine prescription.

There is a risk of developing chronic kidney disease (CKD) post-pregnancy, especially in cases where pregnancy occurs early after transplantation or where pre-existing renal impairment is present. Therefore, the timing of pregnancy relative to transplantation is crucial, with recommendations generally advising a delay of at least one to two years post-transplant before attempting conception [58].

Pregnancy in kidney transplant recipients is feasible but requires a multidisciplinary approach to minimize risks. Pre-conception planning, careful adjustment of immunosuppressive therapy, and close monitoring during pregnancy are essential to optimize maternal and fetal outcomes. With appropriate management, most women with a kidney transplant can achieve a successful pregnancy.

## 8. Conclusions

Nephrological disorders during pregnancy pose significant challenges due to their complex interplay with maternal physiology and fetal development. Timely recognition and appropriate management of these conditions are critical to optimizing outcomes for both the mother and fetus. Conditions such as chronic kidney disease, preeclampsia, and acute kidney injury may present with overlapping features but require distinct management approaches. Moreover, pregnancy-specific pathologies such as HELLP syndrome, thrombotic microangiopathies, and acute fatty liver of pregnancy can lead to rapid clinical deterioration if not promptly addressed. The table below (Table 2) summarizes key nephrological entities in pregnancy, outlining their associated risks and recommended interventions to guide clinical decision-making.

## Figures and Tables

**Table 1:** Management of pregnancy-specific causes of acute kidney injury (AKI).

Management of AKI in Pregnancy	
Pregnancy-Specific Causes of AKI	Management
HELLP	Blood pressure control Prevention of eclamptic seizures (e.g., magnesium sulfate) Avoid liquid overload Mode of delivery according to gestational age
TTP or HUS	Plasma exchange Fresh frozen plasma (if plasma exchange unavailable) Corticosteroids (particularly in TTP) Rituximab (in refractory or relapsing cases) Eculizumab (especially in atypical HUS)
Acute hepatic steatosis in pregnancy	Prompt delivery of the fetus Management of hypoglycemia Coagulopaty correction Management of hepatic encephalopathy Treatment of diabetes insipidus Acute liver failure management
Ureteral obstruction secondary to gravid uterus	Ureteral stent placement Percutaneous nephrostomy Delivery if indicated

**Table 2:** Summary of key nephrological conditions in pregnancy: risks and management.

Condition	Associated Maternal/Fetal Risks	Recommended Interventions
Chronic Kidney Disease (CKD)	Worsening renal function Preeclampsia Fetal growth restriction Preterm birth	Blood pressure control Close monitoring of renal function Low-dose aspirin for preeclampsia prevention Early referral to nephrology
Preeclampsia/HELLP Syndrome	AKI Liver dysfunction Coagulopathy Eclampsia Fetal compromise or demise	Antihypertensive therapy Magnesium sulfate for seizure prophylaxis Timely delivery based on maternal/fetal status
Acute Kidney Injury (AKI)	Maternal fluid/electrolyte imbalance Need for dialysis Fetal hypoxia or demise	Treat underlying cause Optimize fluid balance Renal support if indicated (e.g., dialysis)
Thrombotic Microangiopathy (TTP/HUS)	Thrombocytopenia Microangiopathic hemolytic anemia Multiorgan failure Intrauterine fetal demise	Plasma exchange Corticosteroids Eculizumab (aHUS) Supportive care Multidisciplinary management
Acute Fatty Liver of Pregnancy (AFLP)	Hepatic failure Hypoglycemia DIC AKI High maternal and fetal mortality	Immediate delivery irrespective of gestational age Supportive care for liver/kidney dysfunction ICU monitoring
ADPKD	Hypertension UTIs Infected cysts Preeclampsia Possible intracranial aneurysm rupture	Blood pressure control UTI surveillance/treatment Genetic counseling Low-dose aspirin for preeclampsia prevention
Lupus Nephritis	Flare risk Preeclampsia Fetal growth restriction Preterm delivery	Preconception disease control Immunosuppression (e.g., azathioprine, hydroxychloroquine) Low-dose aspirin Frequent monitoring

## Data Availability

No new data were created or analyzed in this study. Data sharing is not applicable to this article.
